# Regulation of follistatin-like 3 expression by miR-486-5p modulates gastric cancer cell proliferation, migration and tumor progression

**DOI:** 10.18632/aging.203412

**Published:** 2021-08-23

**Authors:** Zhou-Tong Dai, Yuan Xiang, Xiao-Yu Zhang, Qi-Bei Zong, Qi-Fang Wu, You Huang, Chao Shen, Jia-Peng Li, Sreenivasan Ponnambalam, Xing-Hua Liao

**Affiliations:** 1 Institute of Biology and Medicine, College of Life and Health Sciences, Wuhan University of Science and Technology, Hubei 430081, P.R. China; 2 Department of Medical Laboratory, Central Hospital of Wuhan, Tongji Medical College, Huazhong University of Science and Technology, Wuhan 430014, P.R. China; 3 School of Molecular and Cellular Biology, University of Leeds, Leeds LS2 9JT, United Kingdom

**Keywords:** follistatin, FSTL3, gastric cancer, miR-486-5p, cell proliferation

## Abstract

Cancer development and progression can be regulated by the levels of endogenous factors. Gastric cancer is an aggressive disease state with poor patient prognosis, needing the development of new diagnostics and therapeutic strategies. We investigated the close association between follistatin-like 3 (FSTL3) and different cancers, and focused on its role in gastric cancer cell function. Using cancer bioinformatics, we found that FSTL3 expression is elevated in a large majority of the 33 cancers we analyzed in publicly available cancer databases. Elevated levels of FSTL3 is associated with poor patient prognosis in gastric cancer. In a comparison of normal gastric epithelial cells and gastric cancer cell lines, FSTL3 expression was consistently elevated in gastric cancer cells. Overexpression of FSTL3 promoted gastric cancer cell viability, proliferation and migration. Conversely, FSTL3 knockdown inhibits these cellular processes. Using bioinformatics, we found that the *FSTL3* mRNA has a potential binding site in the 3’-UTR for a small microRNA, miR-486-5p. Further bioinformatics revealed significant negative correlation between FSTL3 and miR-486-5p levels. Using luciferase reporter constructs, we provide evidence that the 3’UTR from the *FSTL3* mRNA can confer downregulation in the presence of miR-486-5p. These studies lead us to conclude that FSTL3 has oncogenic properties and increased expression of this gene product promotes gastric cancer development and progression.

## INTRODUCTION

Globally, gastric cancer is one of the most common malignant cancers with serious consequences for population health and mortality. The 2020 Cancer Statistics Survey shows that the gastric cancer mortality rate is ~40% [1]. The development and use of gastroscopy has dramatically increased diagnosis and treatment, this is most effective in early stage disease [2–4], whereas most gastric cancer patients present in the latter stages of disease to the clinic. Gastric cancer is associated with significant secondary metastases which cannot be treated by conventional surgery [5]. The prognosis of advanced stage gastric cancer patients is low, with a typical survival period of 8-12 months [6–8]. Gastric cancer tumor infiltration and metastasis are key causes of poor patient prognosis [9]. Mechanistic explanations of such effects include oncogene activation, tumor suppressor inactivation, and abnormal cellular metabolism. The emergence of cancer bioinformatics has led to the rapid identification and validation of new cancer markers and prognostic indicators. For example, Pan and colleagues found that layilin (LAYN) is a new prognostic and immune infiltration marker in gastric cancer [10]. Aberrant expression of the homeobox-related transcription factor HOXC10 is also linked to gastric cancer cell migration, proliferation and tumor development [11]. The application of cancer bioinformatics is thus a powerful tool in identifying new gastric cancer biomarkers and therapeutic targets.

The follistatin family of secreted glycoproteins includes follistatin-like 3 (FSTL3). More than 20 yrs ago, it was discovered that the *FSTL3* locus undergoes chromosomal rearrangement linked to development of non-Hodgkin lymphoma [12]. Subsequent work has implicated FSTL3 in cellular differentiation, insulin resistance, aging, obesity and arteriosclerosis [13–16]. However, recent studies implicated FSTL3 in the regulation of a range of cancers. For example, FSTL3 expression can be used to distinguish between benign and malignant breast cancer states [17]. Moreover, an FSTL3 antagonist inhibited breast cancer cell proliferation [18]. However, how FSTL3 regulates cancer development and progression is unclear; furthermore, very little is known about FSTL3 regulation of gastric cancer.

Non-coding RNAs (ncRNAs) usually do not encode protein but regulate protein translation and/or mRNA degradation [19]. Such ncRNAs include lncRNAs, microRNAs and circRNAs [20]. A microRNA (miRNA) is an endogenously expressed non-coding RNA 18-25 nucleotides in length, which frequently bind to the 3'-untranslated region (3’-UTR) of the target mRNA, causing degradation or a block in translation by ribosomes [21]. Abnormal miRNA expression can affect cancer development and progression by modulating cell proliferation, migration and invasion [22, 23]. The identification of new gastric cancer biomarkers associated with such features of cancer cell behavior could provide new opportunities for disease intervention.

In this study, we used cancer bioinformatics to evaluate FSTL3 levels in different cancers in the TCGA database. Further analysis of FSTL3 levels in normal and tumor gastric tissues was analyzed to evaluate the potential role of FSTL3 as a gastric cancer biomarker. We then addressed the role of FSTL3 in gastric cancer using *in vitro* cellular models alongside *in vivo* animal models. Our studies now provide new insights into how abnormal control of FSTL3 expression regulates gastric cancer development and progression.

## MATERIALS AND METHODS

### Bioinformatics

All patient data used for this analysis were derived from the public databases such as TCGA [24] (https://tcga-data.nci.nih.gov/tcga/) and GEO (https://www.ncbi.nlm.nih.gov/). R software was used to analyze gene expression and perform functional gene enrichment. Moreover, using the visualization tools in StarBase [25] (https://web.archive.org/web/20110222111721/http://starbase.sysu.edu.cn/), we were able to evaluate the link between miRNA levels and clinical cancer datasets in TCGA. The interaction properties between miRNA and mRNA were predicted using StarBase. Protein-protein interaction (PPI) studies was carried out using the STRING database [26] (https://string-db.org). Cytoscape (https://cytoscape.org/) with the MCODE app was used to analyze the PPI core subnet.

### Cell culture

Human gastric epithelial cell line GES1 and human gastric cancer cell lines BGC-823, MGC-803, SGC-7901, AGS, and HGC-27 were purchased from the cell bank at the Chinese Academy of Sciences. MGC-803, SGC-7901, and HGC-27 cells were cultured in RPMI-1640, AGS cells cultured in DMEM/F12 and the remaining cell lines cultured in DMEM (GIBCO, USA). All cells were cultured in medium supplemented with 10% fetal bovine serum (GIBCO, USA), 100 μg/ml penicillin, and 100 μg/ml streptomycin and grown at 37° C in 5% CO_2_.

### Lentiviral transduction

HEK293T cell line were co-transfected with plasmids pLKO.1, pCDH (synthesized by Shanghai Gema Pharmaceutical Technology Co., Ltd.), VSVG, and GAG-POL using Lipofectamine 3000 (Invitrogen, USA), for the production of replication-defective lentiviral particles. Recombinant lentivirus was harvested at either 48 or 72 h after plasmid transfection. Cell medium was concentrated using PEG-8000 and virus titer determined. Viral particles were added to cultured cells and stable clones selected using 1 μg/ml puromycin (Sigma-Aldrich, USA), 2-3 days after lentiviral transduction.

### Western blotting

All proteins were separated by SDS-PAGE and then electroblotted onto PVDF membranes (Millipore, USA) before blocking with 5% (w/v) skimmed milk (BD, USA) for 1 h. The blocked PVDF membrane was incubated with diluted primary antibody in blocking buffer at 4° C overnight. The next day, the PVDF membrane was rinsed and incubated at room temperature with diluted HRP-conjugated secondary antibodies such as anti-mouse-HRP (Abcam, USA) or anti-rabbit-HRP (Abcam, USA) for 2 h. ECL luminescent solution (Meilunbio, China) was used to visualize bound antibodies using a digital gel imaging system (Biorad, USA). The antibodies used are: mouse anti-β-Actin used at 1:5000 (CST, USA; #4970S), FSTL3 used at 1:1000 (Abcam, USA; #ab232761).

### Dual-luciferase reporter assay

Luc-3'-UTR of FSTL3 and mutant form were separately subcloned into the pmirGLO plasmid (Addgene, USA) to establish wt-FSTL3-luc (WT) and mut-FSTL3-luc (Mut) plasmid constructs respectively. The miRNA mimic, internal control, and parental luciferase plasmid were co-transfected into cells. Luciferase activity was assayed 48 h after transfection using the Dual-Luciferase Reporter Assay System (Promega, USA).

### RNA isolation and qRT-PCR

RNeasy Plus Universal Mini Kit (QIAGEN, USA) was used to isolate total RNA from cell lines, and HiScript® II 1^st^ Strand cDNA Synthesis Kit (Vazyme, China) was used to reverse transcribe into cRNA. At the same time, the miRNeasy Micro Kit (QIAGEN, USA) was used to isolate miRNA from cell lines, and the miRNA 1^st^ Strand cDNA Synthesis Kit (Vazyme, China) was used for reverse transcription of miRNA. For the extracted RNA and miRNA, qRT-PCR was performed on the Bio-Rad CFX-96 (Biorad, USA) system using the SYBR Green (Yisen, USA) method to determine the relative RNA levels. β-actin and U6 mRNAs were used as endogenous controls. The primer sequences used to detect *FSTL3* and *Ki67* mRNAs are: FSTL3-F: 5-GTGCCTCCGGCAACATTGA-3, FSTL3-R: 5-GCACGAATCTTTGCAGGGA-3, Ki67-F:5-GGGCCAATCCTGTCGCTTAAT, Ki67-R:5-GTTATGCGCTTGCGAACCT-3. The synthesis of primers, plasmid sequencing, miRNA reverse transcription, sequencing, primer synthesis and FSTL3 siRNA synthesis were done commercially (Ribobio, China).

### RNA immunoprecipitation (RIP)

RNA-protein-antibody complexes were captured using Protein A/G (ThermoFisher, USA). RNA was eluted by adding TRIzol directly to magnetic beads and isolated as per the manufacturer's instructions. cDNA was synthesized using HiScript® II 1^st^ Strand cDNA Synthesis Kit (Vazyme, China) and analyzed by qRT-PCR.

### Cell viability assay

In a 96-well plate, 3×10^3^ cells in a 100 μl volume were added to each well; each group had 5 multiple wells. After allowing the cells to adhere, 10 μl of CCK-8 solution (Dojindo, Japan) was added to each well. The 96-well plate was incubated in an incubator for 1 h, and absorbance measured at 450 nm using a microplate reader (BioTek, USA). The assay was independently repeated three times.

### Cell migration and invasion assay

A Transwell filter or chamber (Corning, USA) was used to analysis cell migration and invasion. 600 μl of cell suspension was added to the upper chamber, and the cells were suspended serum-free medium. Matrigel (BD, USA) was added to the upper chamber for invasion assay or 10% FBS added to the lower chamber for cell migration assay. 24 h after incubation, the cells that had migrated through the Transwell membrane filter were fixed with formalin (GBICO, USA) and stained with crystal violet. The number of migrated cells were imaged using an inverted microscope (Olympus, Japan) and fields of cells counted. At least five random fields of view were selected for each calculation, and all determinations were independently repeated three times.

### Wounded cell monolayer closure assay

An *in vitro* wounded cell monolayer assay was also used to study cell migration. The cells were seeded in 6-well plates (1 × 10^6^ per well), and after cell adhesion, linear scratch wounds were made in the cell monolayer using a sterile 200 μl plastic pipette tip. Photomicrographs were captured using a digital Olympus camera (Olympus, Japan) 48 h after treatment or stimulation.

### Human-mouse tumor xenograft model

Animal experiments were conducted under the guidelines of the Laboratory Animal Center of Wuhan University of Science and Technology. BALB/c Nude mice, which were 4 weeks old and ~15 g weight per mouse were purchased from Beijing Huafukang Experimental Animal Co., Ltd., and housed in the Experimental Animal Center of Wuhan University of Science and Technology. Human gastric cancer cells were digested with trypsin (GIBCO, USA) without phenol red and EDTA, then resuspended in PBS containing 50% Matrigel (BD, USA). The cell suspension 2x10^6^ cells/ml was injected subcutaneously into the dorsal side of the Nude mice. The mice were sacrificed 4 weeks later for biochemical and histopathological analyses were performed on the tumor samples.

To study metastasis *in vivo*, 2×10^5^ cells of each group were re-suspended in 100 μl PBS, and intravenously injected into the tail vein of BALB/c nude mice (10 mice/group). After 28 days, the number of mice with metastasis was counted.

### Histology and histochemistry

The tissues were fixed, dehydrated, and paraffin-embedded in the 10% formalin to make 5 μm tissue sections for hematoxylin-eosin staining, and observation using a digital microscope (Olympus, Japan). Xylene was used to dewax them twice for 15 min each time. The tissue sections were incubated in 3% (v/v) H_2_O_2_ (Sigma-Aldrich, St. Louis, MO, USA) at 37° C for 30 min, boiling in 0.01 M citric acid buffer at 95° C for 20 min, and then blocked with non-specific serum solution at 37° C for 10 min before incubation with diluted primary antibodies at 37° C for 2 h. Tissue sections were then incubated with species-specific secondary antibodies conjugated to horseradish peroxidase (HRP) and counterstained with hematoxylin (Meilunbio, China) at room temperature for 4 min. The sections were observed using a digital microscope (Olympus, Japan). The following antibodies were used: rabbit anti-FSTL3 at 1:50 (Abcam, USA; #ab232761).

### Cell adhesion assay

In a 96-well plate with Matrigel (BD, USA) already spread. 2000 cells were seeded in each well, and serum-free medium was used to cultivate the cells. After 2 h of incubation, cells adhered to Matrigel were washed twice with PBS. After fixation with 4% paraformaldehyde (Meilunbio, China) for 10 min, DAPI (Meilunbio, China) staining was used to observe the number of adherent cells under a fluorescence microscope (Olympus, Japan). Each experiment was repeated at least at least 3 times.

### Focal adhesion assay

The experimental method is like immunofluorescence. The following antibodies were used: anti-F-actin (Meilunbio, China), anti-vinculin (CST, USA #91459).

### Statistical analysis

Comparisons between groups and datasets were performed using R software version 4.0.3. Comparisons were completed using a one-way analysis of variance (ANOVA) and two-tailed Student's t-test. Correlations were calculated using the Pearson correlation. Kaplan–Meier analyses were used to analyze cancer patient survival. All data are presented with error bars indicating mean ±SD. Statistical significance cutoff is at *P*<0.05.

### Availability of supporting data

The data and files generated during this study are available from the corresponding author upon request.

## RESULTS

### FSTL3 expression is associated with gastric cancer and poor patient prognosis

**Figure 1:**
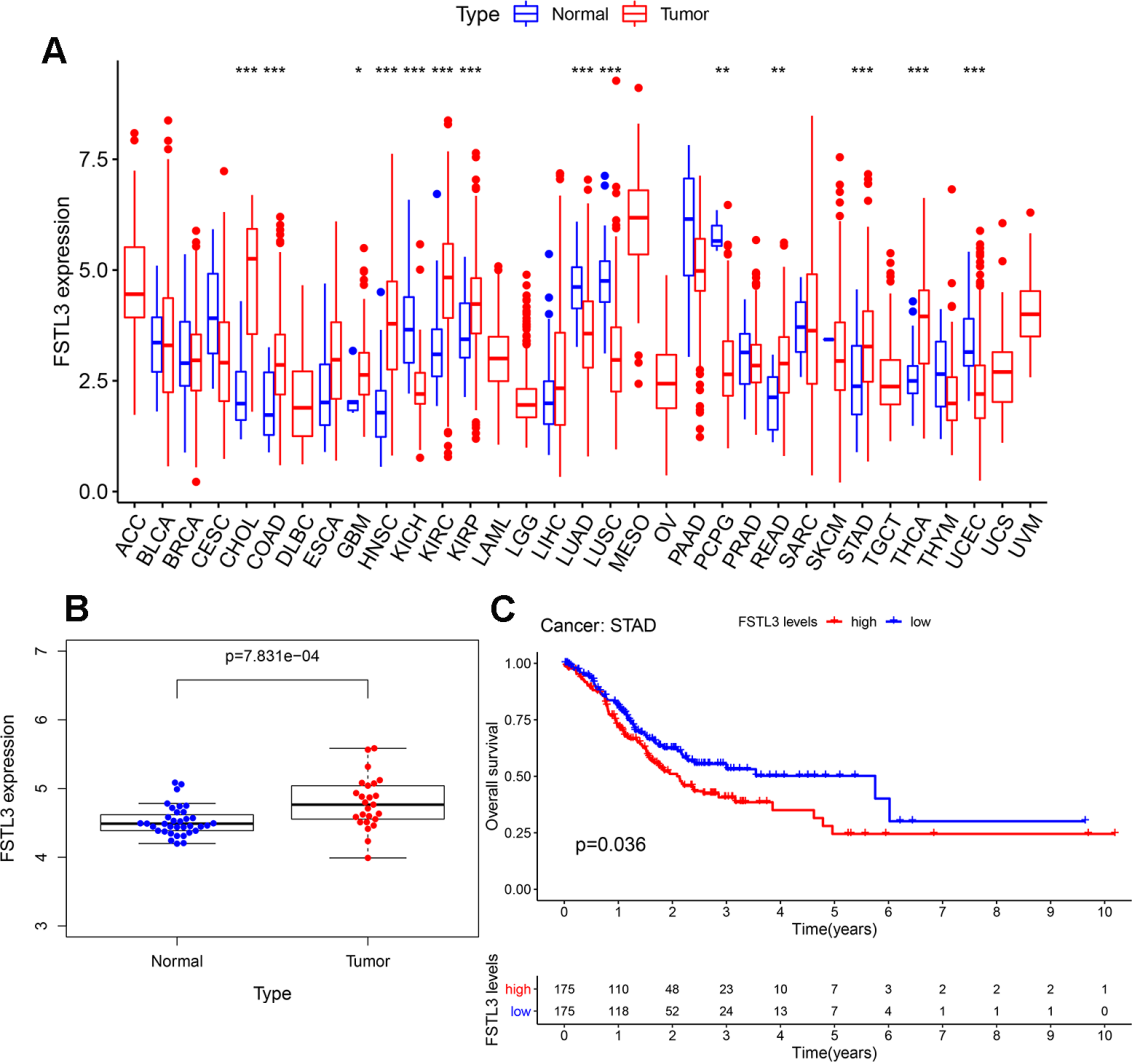
**Elevated FSTL3 expression in gastric cancer is linked to poor patient prognosis.** (**A**) Profiling of FSTL3 expression in 33 cancers within the TCGA database. (**B**) Analysis of FSTL3 expression in non-cancerous and cancerous gastric tissues using the GEO clinical dataset GSE33335. (**C**) Kaplan-Meier analysis of gastric cancer patient overall survival after classification into high (red) and low (blue) FSTL3 expression groups (n=175). Significance indicated as follows: *P<0.05, **P<0.01, ***P<0.001.

FSTL3 expression was profiled in clinical datasets for 33 cancers deposited in the TCGA database (Figure 1A). FSTL3 expression was elevated, notably in CHOL, COAD, GBM, HNSC, KIRC, KIRP, READ, STAD and THCA cancers; however, FSTL3 expression was decreased in KICH, LUAD, LUSC, PCPG, UCEC cancers. To further analyze gastric cancer (stomach adenocarcinoma, STAD) increased FSTL3 expression (Figure 1A) we used the GEO clinical dataset GSE33335. This dataset contains gene profiling data from cancerous tissues and non-cancerous adjacent tissues taken from 25 gastric cancer patients. FSTL3 expression was increased in gastric cancer compared to control tissues (Figure 1B). Next, we evaluated the clinical implications of FSTL3 expression in gastric cancer patient prognosis using Kaplan-Meier analysis (Figure 1C). The median FSTL3 expression in gastric cancer tissues was defined as the cut-off value. All STAD patients were divided into FSTL3 low expression and high expression groups (n=175). We found that the high FSTL3 expression caused decreased STAD patient survival over a 10 yr period, *P*=0.036 (Figure 1C).

We also performed Univariate Cox analysis to analyze histopathological characteristics, age, gender, and disease staging (Table 1). FSTL3 expression was increased with older age and later stages of gastric cancer disease (Table 1). However, disease grade nor gender had no significant correlation with FSTL3 expression (Table 1). Gastric cancer patients thus exhibit increased FSTL3 expression which influences disease progression and survival.

**Table 1:** Univariate Cox analysis of gastric cancer patient clinical features linked to increased FSTL3 expression.

	**HR**	**HR.95L**	**HR.95H**	***P*-value**
**age**	1.026965748	1.007829689	1.046465151	**0.00556017**
**gender**	1.483828302	0.979779065	2.247186647	0.062392212
**grade**	1.367875492	0.946580494	1.976676441	0.095375967
**stage**	1.535478376	1.221185829	1.930659353	**0.000242497**
**T**	1.297541887	1.023344886	1.645207762	**0.031516711**
**M**	2.048306027	1.096196896	3.827375896	**0.024581693**
**N**	1.267206892	1.068895108	1.5023114	**0.006386528**

Bold values are statistically significant (*P*<0.05).

### Gene enrichment and functional analyses for elevated FSTL3 expression

**Figure 2:**
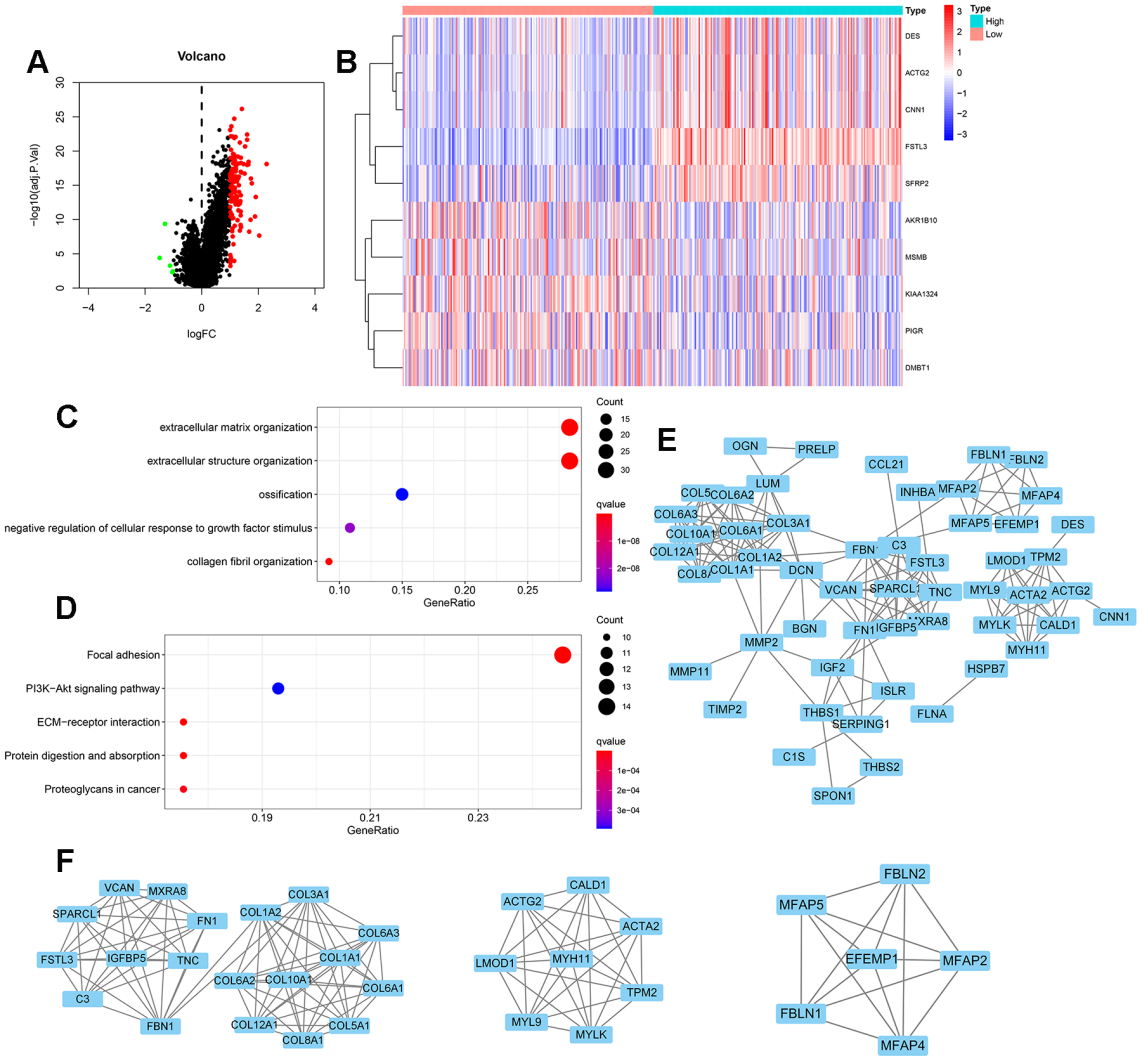
**Differential gene expression and functional enrichment during FSTL3 overexpression.** (**A**) Volcano map of differentially expressed genes under conditions of elevated FSTL3 levels; adjusted *P*<0.05. (**B**) The top 10 differentially expressed genes (linked to FSTL3 overexpression) in gastric cancer disease presented as a gene expression heat map. (**C**) Gene ontology (GO) term enrichment analysis; adjusted *P*<0.05. (**D**) Kyoto Encyclopedia of Genes and Genomes (KEGG) biochemical pathway enrichment analysis; adjusted *P*<0.05. (**E**) Protein-protein interaction (PPI) network analysis for 122 differentially expressed genes linked to gastric cancer; minimum required interaction score: 0.900. (**F**) Specific PPI nodes within the full network with MYOCD tool in Cytoscape.

We screened for differentially expressed genes from the FSTL3 high and low expression groups in gastric cancer (Figure 1C). We found 122 differentially expressed genes linked to the STAD database in TCGA and these are depicted as points on the gene expression log plot (Figure 2A). The top 10 differentially expressed genes linked to FSLT3 expression are shown in Figure 2B. These differentially expressed genes analyzed by gene ontology descriptors include extracellular matrix organization, extracellular structure organization, ossification, negative regulation of cellular responses to growth factor stimulus and collagen fibril organization (Figure 2C). The top five biochemical pathways identified by enrichment analysis using the Kyoto Encyclopedia of Gene and Genomes (KEGG) are focal adhesions, PI3K-Akt signal transduction, ECM-receptor interactions, protein digestion and absorption, and cancer proteoglycans (Figure 2D). These 122 differentially expressed genes were imported into the STRING database to construct PPI networks. These genes can be linked into a singular network with 121 nodes and 173 edges (Figure 2E). COL1A2, COL3A1, COL1A1, COL5A1, COL5A1 constitute the top five nodes. By using the MYOCD tool in Cytoscape, we identified core subnet modules within this PPI network; the top three nodes are depicted (Figure 2F).

### Elevated FSTL3 levels promote gastric cell proliferation and migration

**Figure 3:**
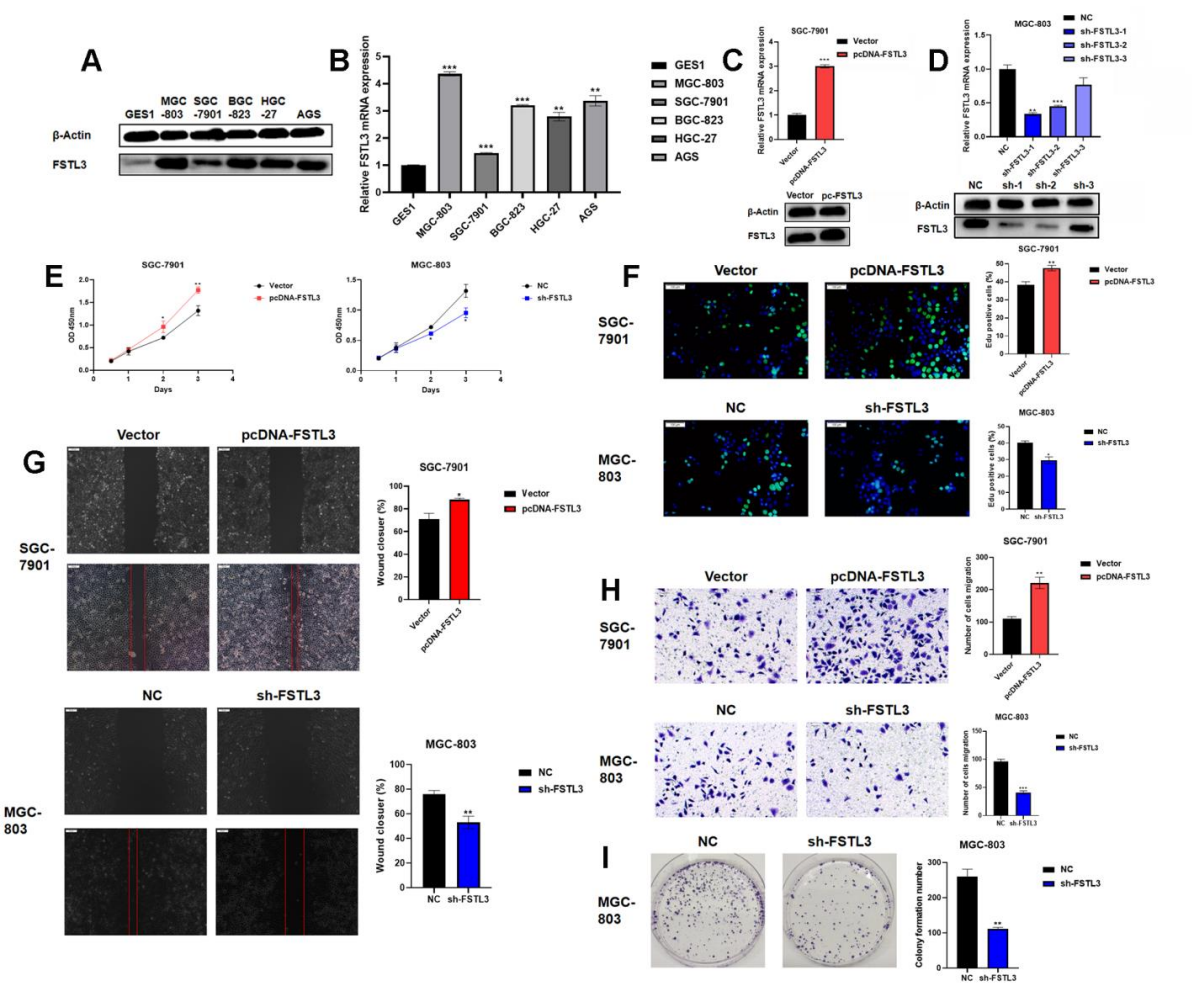
**FSTL3 overexpression or knockdown modulates gastric cancer cell viability, proliferation and migration.** (**A**) Western blot analysis of FSTL3 protein levels in GES1, MGC-803, SGC-7901, BGC-823, HGC-27 and AGS gastric cell lines; blotting for the housekeeping protein β-actin was used as a loading control in this experiment. (**B**) Analysis of relative *FSTL3* mRNA levels in GES1, MGC-803, SGC-7901, BGC-823, HGC-27 and AGS gastric cell lines using qRT-PCR (see Materials and Methods). (**C**) Overexpression of FSTL3 in gastric cancer cell line SGC-7901 monitored by Western blotting and qRT-PCR. (**D**) FSTL3 knockdown in gastric cancer cell line MGC-803 monitored by Western blotting and qRT-PCR. (**E**) FSTL3 overexpression (SGC-7901) or knockdown (MGC-803) cell lines were analyzed for cell viability compared to controls. (**F**) FSTL3 overexpression (SGC-7901) or knockdown (MGC-803) cell lines were assayed for cell proliferation using EdU incorporation. (**G**) Wounded cell monolayer assays on FSTL3 overexpression (SGC-7901) or knockdown (MGC-803) cell lines (48h). (**H**) Transwell cell migration assay on FSTL3 overexpression (SGC-7901) or knockdown (MGC-803) cell lines followed by staining of Transwell filters to detect migrated cells. (**I**) FSTL3 knockdown and effects on MGC-803 cell colony formation. Quantification in panels (**B**–**I**) were carried out as described in Materials and Methods and compared to control cell lines. Error bars indicate +SEM; significance indicated by asterisks, *P<0.05, **P<0.01, ***P<0.001; number of experiments, n=3.

The expression of FSTL3 in a range of gastric cell lines was evaluated (Figure 3). Normal gastric epithelial cell line GES1 was compared to 5 different gastric cancer cell lines and Western blotting of cell lysates showed clear differences in FSTL3 protein levels (Figure 3A). FSTL3 expression was lowest in control GES1 cell line; however, FSTL3 levels were raised in all gastric cancer cell lines with the highest expression in MGC-803 and lowest in SGC-7901 cell lines (Figure 3A). Further analysis of the endogenous *FSTL3* mRNA levels using qRT-PCR further supported this trend, with SGC-7901 showing 1.5-fold rise compared to 4-fold rise in MGC-803 cells (Figure 3B). These 2 lines were then selected as low and high FSTL3 expressors for testing FSTL3 regulation of gastric cancer cell function. In order to explore whether FSTL3 regulates gastric cancer cell function, we overexpressed FSTL3 in SGC-7901 cells (Figure 3C). We showed that this FSTL3 plasmid caused 3-fold rise in FSTL3 mRNA levels; furthermore, this correlated with an increase in FSTL3 protein levels (Figure 3C). We then made a lentiviral system to express small hairpin RNAs (shRNAs) that target the *FSTL3* mRNA; screening 3 different *FSTL3*-specific shRNAs showed that sh-1 was most effective (Figure 3D). *FSTL3* mRNA levels were reduced by 60% using sh-1; a similar knockdown in FSTL3 protein levels was also evident (Figure 3D).

We then assessed FSLT3 overexpression or knockdown on gastric cancer cell responses (Figure 3E–3H). Measurement of cell viability showed increased FSTL3 levels promoted gastric cancer cell line SGC-7901 viability; in contrast, FSTL3 knockdown caused decreased cell viability in MGC-803 (Figure 3E). Measurement of new DNA synthesis using EdU incorporation showed 25% increased cell proliferation upon FSTL3 overexpression in gastric cancer cell line SGC-7901 (Figure 3F). Again, FSTL3 knockdown caused 25% decrease in MGC-803 cell proliferation (Figure 3F). We then asked whether FSTL3 overexpression or knockdown affected cell monolayer closure, which is dependent on both cell proliferation and migration (Figure 3G). FSTL3 overexpression promoted a 20% increase in monolayer wound closure compared to control in gastric cancer cell line SGC-7901 (Figure 3G). However, FSTL3 knockdown promoted a 30% decrease in monolayer wound closure compared to control in gastric cancer cell line MGC-803 (Figure 3G).

We also assessed FSTL3 overexpression or knockdown on cell migration using the Transwell assay (Figure 3H). FSTL3 overexpression promoted a 2-fold increase in cell migration compared to control in gastric cancer cell line SGC-7901 (Figure 3H). However, FSTL3 knockdown promoted a 60% decrease in cell migration compared to control in gastric cancer cell line MGC-803 (Figure 3H). We also assessed FSTL3 to the formation of cell colonies in culture, an important feature of tumor growth (Figure 3I). FSTL3 knockdown caused 50% decrease in gastric cancer cell line MGC-803 colony formation compared to control (Figure 3I). FSTL3 was also overexpressed in the normal gastric epithelial cell line, GES1 (Supplementary Figure 1A). This showed 2-fold increase in *FSTL3* mRNA (Supplementary Figure 1B) and protein (Supplementary Figure 1A) levels. Increased FSTL3 expression promoted increased GES1 cell viability (Supplementary Figure 1C). Analysis of EdU incorporation showed 30% increase in new DNA synthesis in FSTL3-overexpressing cells compared to control (Supplementary Figure 1D). Analysis of GES1 FSTL3-overexpressing cells revealed 75% increase in colony formation compared to control (Supplementary Figure 1E). To further test inference from our bioinformatics studies, we knocked down and overexpressed the expression of FSTL3 in gastric cancer cells. It was found that the over-expression of FSTL3 promotes cell adhesion and the number of focal adhesion spots (Supplementary Figure 2A, 2B).

### FSTL3 promotes gastric cancer cell tumorigenicity *in vivo*

**Figure 4:**
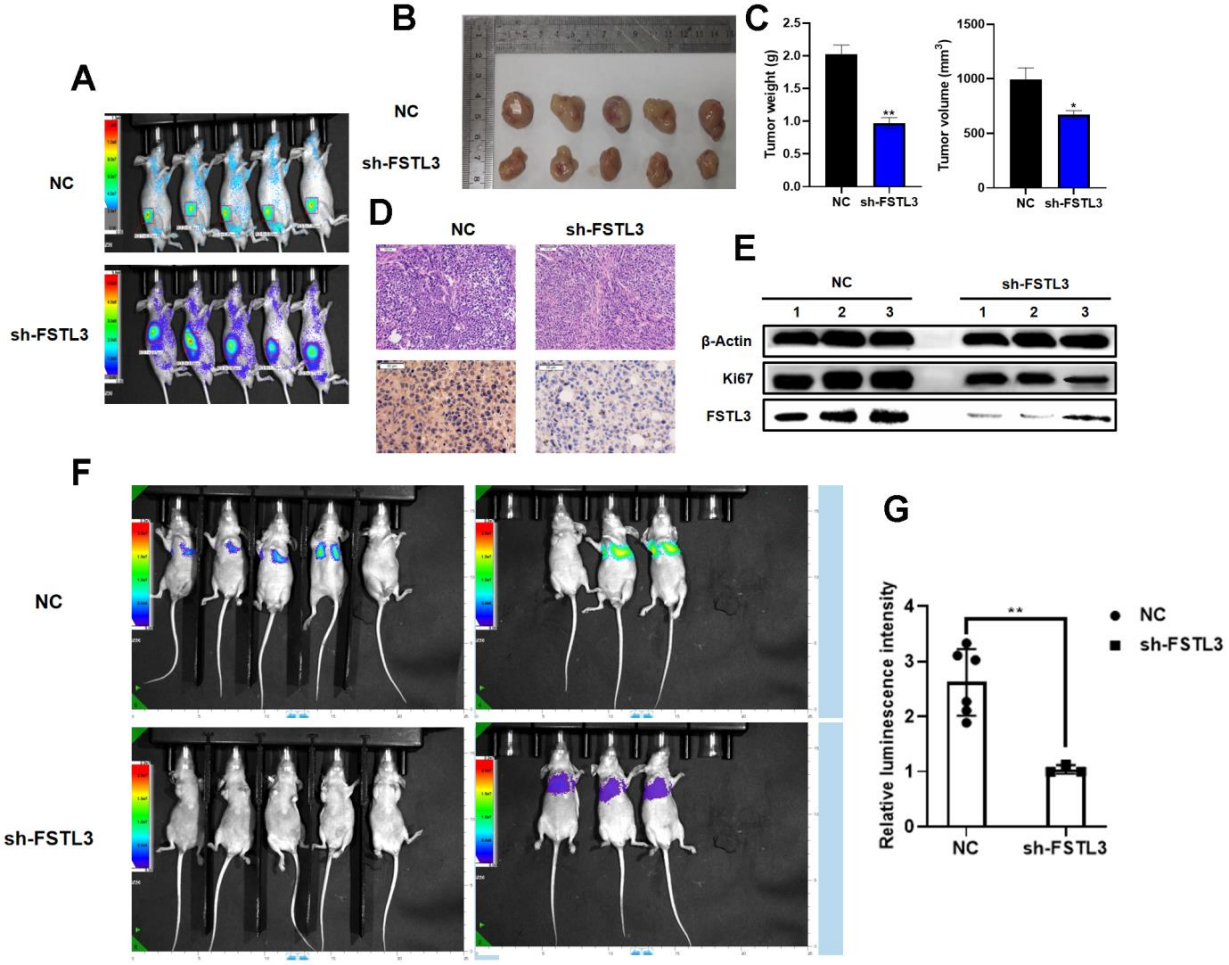
**FSTL3 expression promotes gastric cancer tumorigenicity *in vivo*.** (**A**) Bioimaging of MGC-803-derived tumors in the flanks of Nude mice. Growth of tumors in Nude mice injected for control MGC-803 cells (NC) or cells expressing shRNA-specific for FSTL3 (sh-FSTL3). (**B**) Excision and viewing of subcutaneous tumors derived from Nude mice injected for control MGC-803 cells (NC) or cells expressing shRNA-specific for FSTL3 (sh-FSTL3). (**C**) Analysis of weight or volume of tumors derived from Nude mice injected for control MGC-803 cells (NC) or cells expressing shRNA-specific for FSTL3 (sh-FSTL3). (**D**) Hematoxylin-eosin (upper panels) or Ki67 immunohistochemistry (lower panels) on tissue sections from subcutaneous tumors derived from Nude mice injected for control MGC-803 cells (NC) or cells expressing shRNA-specific for FSTL3 (sh-FSTL3). (**E**) Analysis of FSTL3 and Ki67 using Western blotting of tumors from Nude mice injected for control MGC-803 cells (NC) or cells expressing shRNA-specific for FSTL3 (sh-FSTL3). Blotting of tumor lysates using antibodies to FSTL3 or Ki67; antibodies to β-actin were used to check for protein loading in tumor lysates. (**F**) Bioluminescence imaging results of the lung metastasis frequency from Nude mice injected for control MGC-803 cells (NC) or cells expressing shRNA-specific for FSTL3 (sh-FSTL3). (**G**) Statistical analysis of luminescence intensity. Error bars indicate +SEM; significance indicated by asterisks, *P<0.05, **P<0.01, ***P<0.001; number of experiments, n=3.

We then assessed the contribution of FSTL3 to gastric cancer cell tumorigenicity using an *in vivo* animal model (Figure 4). The MGC-803 gastric cancer cell line expressing the sh-FSTL3 construct with stable FSTL3 knockdown was compared to the parental cell line (control) for the ability to promote tumor development in the immunocompromised Nude mouse model. Bioimaging of MC-803-derived tumors in Nude mice showed that tumor size was reduced (Figure 4A). Tumors were excised and examined visually: again, FSTL3 knockdown suggested smaller tumor size compared to parental MGC-803-derived tumors (Figure 4B). Further analysis revealed 50% reduction in tumor weight upon FSTL3 knockdown (Figure 4C). There was 30% reduction in tumor volume upon FSTL3 knockdown (Figure 4C). Hematoxylin-eosin staining of tumor sections suggested a more irregular cell shape and packing in tumors from the parent cell line MGC-803-derived tumor compared to FSTL3 knockdown conditions (Figure 4D). Analysis of the cell proliferation marker, Ki67, showed a much higher immunoreactivity for nuclear Ki67 in parent cell line MGC-803-derived tumors compared to FSTL3 knockdown tumors (Figure 4D). We then probed for FSTL3 expression in these tumors using Western blotting, and found 70% decrease in *FSTL3* mRNA levels correlated with decreased FSTL3 protein levels in MGC-803 knockdown cells (Figure 4E). Under these condition, stable FSTL3 knockdown caused 30% decrease in expression of cell proliferation marker, Ki67 (Figure 4E).

We then assessed the effects on tumor metastasis *in vivo* using tail vein administration of MGC-803 parental line or MGC-803/sh-1 cell into Nude mice. After allowing tumors to develop and spread systemically, we analyzed the frequency of mouse lung tissues for secondary tumor metastases. There were both 2 deaths in the control group and FSTL3 knockdown group of nude mice. Bioluminescence imaging results revealed that FSTL3 knockdown significantly reduced incidence in the lung of metastatic secondary tumors (Figure 4F, 4G). The expression levels of FSTL3 thus influences both cell proliferation and tumor progression.

### *FSTL3* mRNA is a target of miR-486-5p

**Figure 5:**
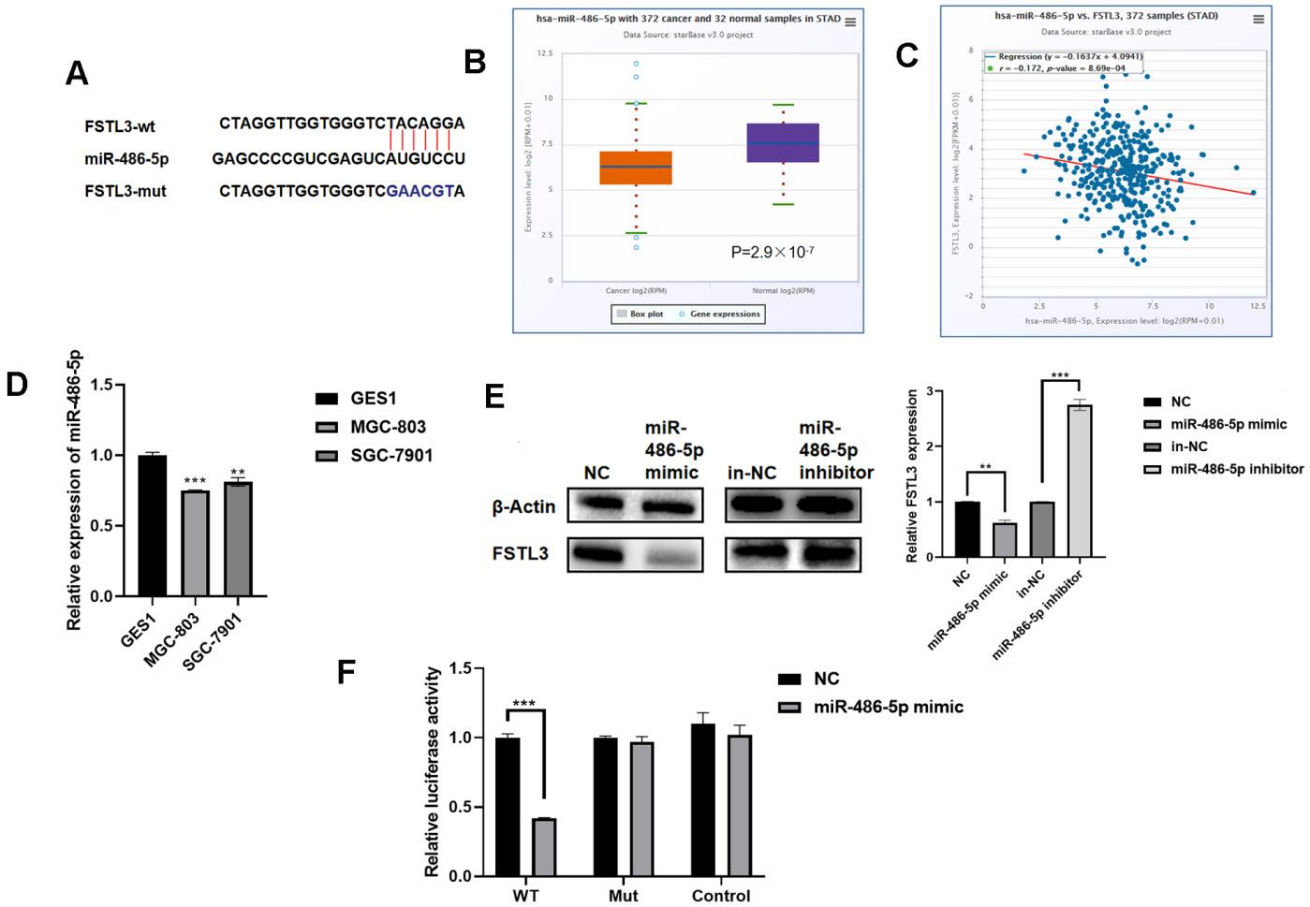
***FSTL3* mRNA is regulated by miR-486-5p levels.** (**A**) Schematic depiction of the predicted binding site for miR-486-5p in the *FSTL3* mRNA. (**B**) Analysis of miR-486-5p levels in gastric cancer tumors vs. normal tissues in the STAD database in TCGA. (**C**) Correlative analysis of miR-486-5p and FSTL3 levels in gastric cancer tumors using the STAD database. (**D**) Profiling of miR-486-5p levels in normal and cancer gastric cancer cell lines using qRT-PCR. (**E**) Western blotting was used to analyze protein and RNA levels in gastric cancer MGC-803 cells subjected to transfection with miR-486-5p mimic or miR-486-5p inhibitor. (**F**) A dual luciferase assay was carried out using transfection of luciferase plasmid constructs alongside miR-486-5p mimic or miR-486-5p inhibitor. HEK 293T cells co-transfected with Luc-FSTL3-wt (WT) or Luc-FSTL3-mut (MUT) plasmids with synthetic miR-486-5p constructs. Error bars indicate +SEM; significance indicated by asterisks, *P<0.05, **P<0.01, ***P<0.001; number of experiments, n=3.

To evaluate whether mRNA turnover was a potential regulatory mechanism to control FSTL3 protein levels, we used StarBase (https://web.archive.org/web/20110222111721/http://starbase.sysu.edu.cn/) to look for potential miRNAs that bind to *FSTL3*. Using this approach, we identified an miRNA termed miR-486-5p as a potential regulator which binds to a site in the 3’UTR of the *FSTL3* mRNA (Figure 5A). To assess the correlation between miR-486-5p, *FSTL3* and cancer status, we used the STAD database within TCGA. We found that miR-486-5p levels were significantly decreased in gastric cancer patients compared to controls (Figure 5B). Further analysis showed a correlation between decreased miR-486-5p levels with increased *FSTL3* levels (Figure 5C). We then examined miR-486-5p levels in normal gastric epithelial cells (GES1) vs. gastric cancer cell lines, MGC-803 and SGC-7901, using qRT-PCR (Figure 5D). There was a significant 20-30% decrease in miR-486-5p levels in gastric cancer cell lines (MGC-803, SGC-7901) compared to normal gastric cells (GES1).

To further verify whether *FSTL3* is indeed a target of miR-486-5p we constructed a synthetic miR-486-5p mimic. Transfection of this miR-486-5p mimic was transfected into gastric cancer cell line MGC-803: FSTL3 expression was decreased 50% (Figure 5E). We also made a synthetic miR-486-5p inhibitor, and delivery of this into gastric cancer cells caused a 1.5-2-fold rise in FSTL3 protein levels; this correlated with a 2.5-fold rise in *FSTL3* mRNA levels (Figure 5E). We also used a dual luciferase reporter assay where the luciferase cDNA was fused to either the wild-type *FSLT3* 3’UTR (FSLT3-wt) or a mutant *FSLT3* 3’UTR that was defective for binding miR-486-5p. Co-transfection of miR-486-5p mimic and luciferase FSTL3-wt showed 60% reduction in luciferase activity (Figure 5F). In contrast, co-transfection of miR-486-5p mimic and luciferase FSTL3-mut showed no effect on luciferase activity (Figure 5F). These data suggest that miR-486-5p directly regulates *FSTL3* mRNA stability and/or degradation.

### MiR-486-5p specificity for *FSTL3* mRNA and effects on gastric cancer cell responses

**Figure 6:**
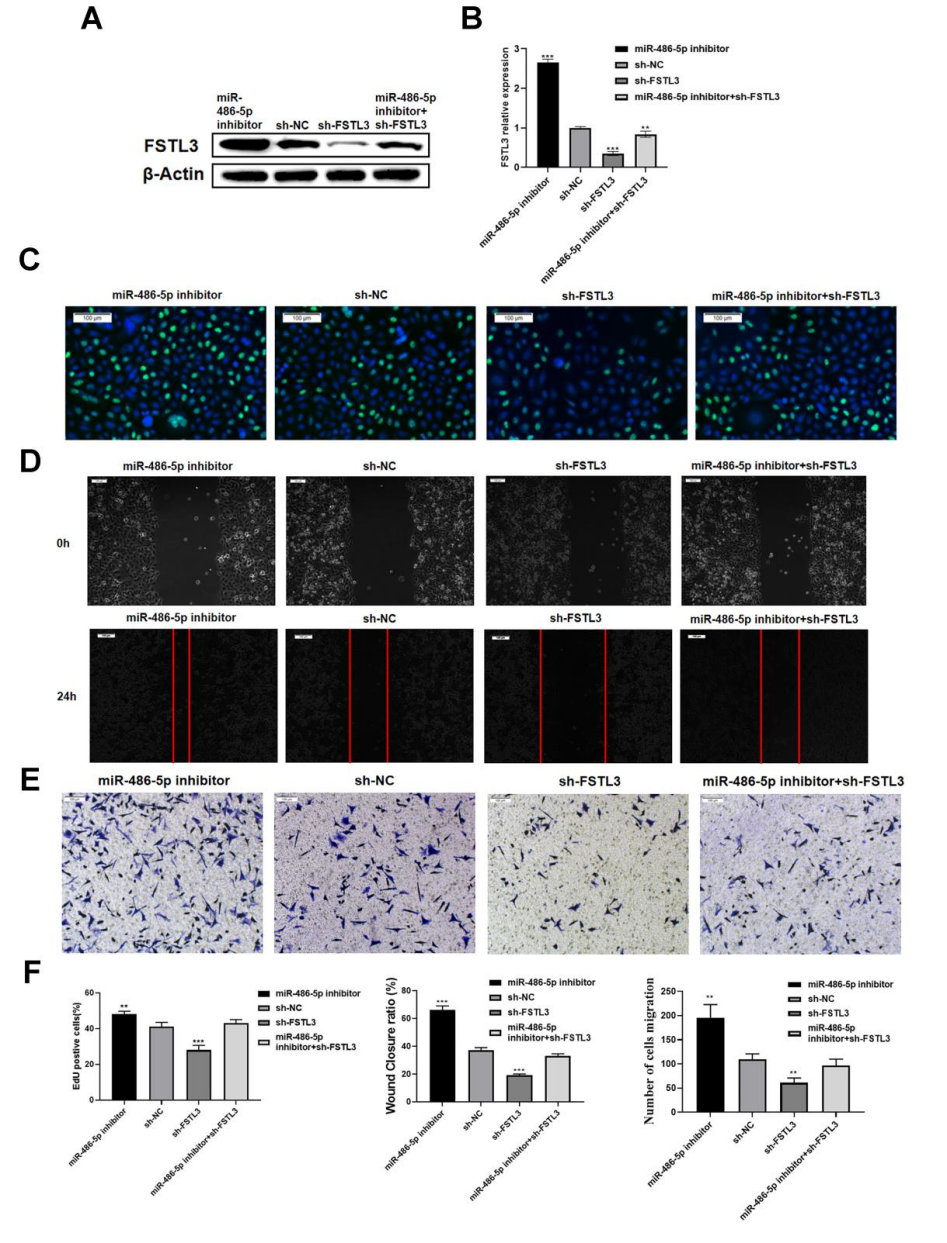
**Evaluating miR-486-5p specificity for *FSTL3* mRNA.** (**A**) Western blotting evaluation of FSTL3 protein levels after transfection of sh-FSTL3 and/or miR-486-5p inhibitor into MGC-803 gastric cancer cells. Monitoring β-actin levels was used as an internal control in these blots. (**B**) Western blot results were quantified by using Image J software. Error bars indicate +SEM; significance indicated by asterisks, **P<0.01, ***P<0.001; number of experiments, n=3. (**C**) Transfection of sh-FSTL3 and/or miR-486-5p inhibitor into MGC-803 gastric cancer cells and evaluation of cell proliferation using EdU assay (24h). (**D**) Wounded cell monolayer closure assay after transfection of sh-FSTL3 and/or miR-486-5p inhibitor followed by imaging at 8 and 24 h post-wounding. (**E**) The Transwell assay for cell migration after transfection of sh-FSTL3 and/or miR-486-5p inhibitor was into MGC-803 cells. Sh-NC construct (shRNA) is a negative control for FSTL3 knockdown. (**F**) Quantified results of EdU, wound healing and transwell migration assays. Sh-NC was analyzed as a control.

To understand the link between *FSTL3* mRNA and miR-486-5p, we evaluated whether such potential regulation impacts on gastric cancer cell responses. We focused on the miR-486-5p inhibitor and its ability to modulate FSTL3 knockdown by shRNA (Figure 6). We transfected alone or co-transfected the shRNA specific for FSTL3 and/or miR-486-5p inhibitor in the MGC-803 cell line followed by Western blotting (Figure 6A). The FSTL3 protein levels were knocked down by sh-FSTL3, however, this effect could be partially reversed by miR-486-5p inhibitor (Figure 6A, 6B). The miR-486-5p inhibitor caused a substantial 2-fold rise in *FSTL3* mRNA levels (Figure 6B). The sh-FSTL3 construct caused 60% knockdown of *FSTL3* mRNA levels; however, this was partially reversed by miR-486-5p inhibitor with 2-fold rise in *FSTL3* mRNA levels (Figure 6B).

We then assessed the effects of targeting miR-486-5p on MGC-803 gastric cancer cell responses. The miR-486-5p inhibitor caused a substantial rise in new proliferating cells compared to control (NC) cells, shown by EdU staining (Figure 6C). The sh-FSTL3 construct caused reduced nuclear EdU staining; however, this was reversed by miR-486-5p inhibitor (Figure 6C). Using the wounded cell monolayer closure assay, we demonstrated that control (NC) cells show wounded monolayer closure after 24 h (Figure 6D). Transfection of miR-486-5p inhibitor caused a dramatic increase in wounded monolayer closure; this miR-486-5p inhibitor caused a partial reversal in the inhibitory effects mediated by sh-FSTL3 construct in this assay (Figure 6D). Similar conclusions were reached in the analysis of cell migration using the Transwell assay (Figure 6E). Transfection of miR-486-5p inhibitor caused a dramatic increase in MGC-803 cell migration; furthermore, miR-486-5p inhibitor and sh-FSTL3 treatment together caused a partial reversal of the inhibitory effects on MGC-803 cell migration caused by sh-FSTL3 construct treatment alone (Figure 6D). Such findings further support the function role of miR-486-5p inhibitor in controlling FSTL3 levels and gastric cell responses.

## DISCUSSION

The follistatin (FST) family of secreted glycoproteins are increasingly associated with different types of cancers. In a functional analysis of a family member, termed FSTL3, we provide evidence that a mechanism which directly modulates *FSTL3* mRNA levels directly impacts on gastric cancer cell responses which contribute to cancer development and progression. This is based on 4 lines of evidence. First, cancer bioinformatics shows a clear association with increased FSTL3 expression in gastric cancer patients. Furthermore, high FSTL3-expressing gastric cancer patients have reduced survival over a 10 yr period. Second, modulation of FSTL3 expression in a human-mouse tumor xenograft model, suggests that higher levels of FSTL3 are linked to increased tumor growth and spread. Third, modulation of FSTL3 shows a clear link with gastric cell responses such as cell viability, proliferation and migration. Finally, *FSTL3* mRNA is regulation by miR-486-5p modulates FSTL3 expression and gastric cancer cell-associated responses.

FSTL3 is a secreted glycoprotein encoded by a gene located on human chromosome 19q13.3. *FSTL3* was originally discovered as a rearranged gene locus associated with some blood disorders and malignancies [12]. The follistatin (FST) family members are single-chain monomeric glycoproteins which potentially interact with other soluble factors and/or membrane receptors [27]. Follistatin can bind to members of the TGF-β superfamily (e.g. activin); it can also bind FSH and inhibits its biological activity. Another family member, FSTL1 is associated with reduced levels in prostate cancer; FSTL1 expression correlates with inflammatory factors and transforming factors [28]. In gastric cancer, it was reported that FSTL1 knockdown promotes apoptosis via the STAT6 signaling pathway [29]. In hepatocellular carcinoma, FSTL5 expression inhibits the HCC growth *in vitro* by inducing apoptosis [30].

An miRNA is a single-stranded non-coding RNA (18-25 nucleotides) endogenously encoded which regulates protein translation or mRNA degradation via interaction with the 3'or 5' untranslated regions (UTRs) of target mRNAs [31]. Many cell biological processes such as cell proliferation, growth, differentiation, apoptosis and the cell cycle are regulated by a variety of miRNAs [32]. miR-486-5p is a recently discovered miRNA which displays abnormal expression in colon cancer [33], gastric cancer [34], liver cancer [35] and other malignant tumors; miR-486-5p levels are linked to tumor growth and invasion. In gastric adenocarcinoma, miR-486-5p is a potential tumor suppressor. Comparison of normal gastric epithelial cells and tissues vs. gastric cancer cell lines and tumors, and is miR-486-5p is a combinatorial risk factor alongside olfactomedin-4 (OLFM4) and fibroblast growth factor 9 (FGF9). The combination of FGF9 expression alongside other genes can inhibit gastric cancer cells proliferation and is an independent risk factor for predicting gastric cancer patient survival [34, 36]. In this study, we found that by combining *in silico* bioinformatics with cellular experiments, miR-486-5p levels were down-regulated in gastric cancer cell lines and tumors. This is supported by previous studies elsewhere [34]. We further delineated the potential site of interaction between miR-486-5p with the 3’UTR of the *FSTL3* mRNA. Overexpression of a synthetic miR-486-5p mimic causes FSTL3 down-regulation; however, use of a synthetic miR-486-5p inhibitor causes a rise in FSTL3 levels. Finally, miR-486-5p and FSTL3 levels correlate negatively in an analysis of gene expression in gastric cancer tissues.

What is the functional target of FSTL3? One possibility is that FSTL3 binds to a cell surface receptor which stimulates autocrine signaling and cellular responses linked to cell proliferation and migration. Alternatively, as noted for the FST founding family member capacity to bind other secreted factors, FSTL3 may sequester autoinhibitory factors thus effecting a net autocrine stimulatory effect. Our findings provide a new framework to better understand gastric cancer development and progression and develop new therapeutic strategies. However, there are also specific limitations of our study. First, the incidence of Asian and African patients within the TCGA database is low, with skewed view of the molecular events underlying cancer processes. Whether FSTL3 is a new diagnostic biomarker of gastric cancer requires the collection of more unbiased clinical datasets across the global population. Next, *FSTL3* mRNA may be the target of multiple miRNAs, and more work is needed to assess such factors. The role of miR-486-5p as a regulator of *FSTL3* miRNA degradation needs to be checked for its ability to target other cellular mRNA species. Our work clearly supports a role for FSTL3 in promoting gastric cancer cell proliferation and migration; furthermore, FSTL3 expression contributes to gastric tumor growth, invasion and metastasis. FSTL3 shows promise as a new biomarker for gastric cancer diagnosis and is a new therapeutic target in this disease.

## Supplementary Material

Supplementary Figures
