# Supplementary material for: Regulation of follistatin-like 3 expression by miR-486-5p modulates gastric cancer cell proliferation, migration and tumor progression
Source: Aging (Albany NY). 2021 Aug 23;13(16):20302–18. doi: 10.18632/aging.203412 (PMC8436905; doi:10.18632/aging.203412)
Supplement: Supplementary Figures [file aging-13-203412-s001.pdf]

SUPPLEMENTARY FIGURES

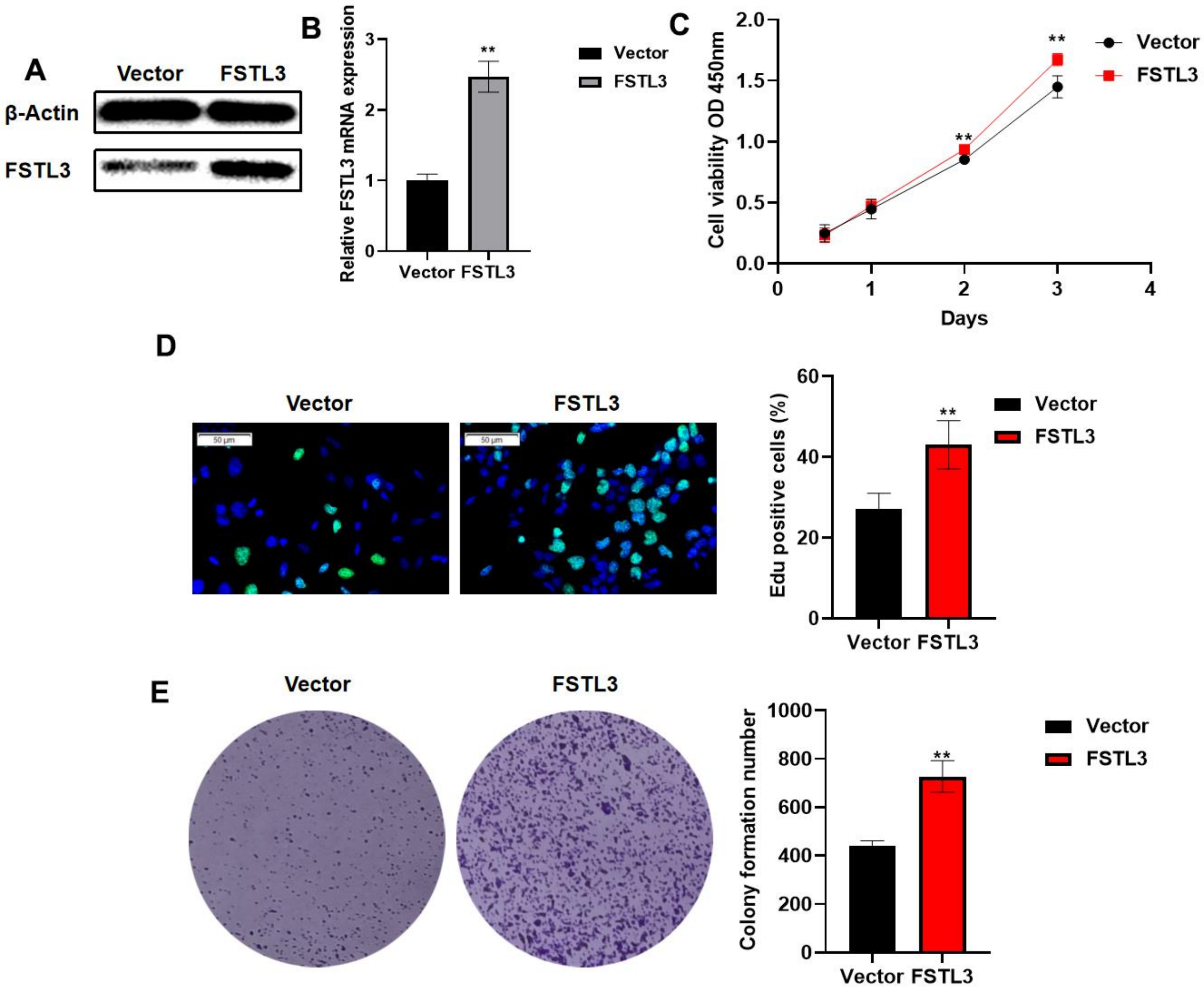

Supplementary Figure 1. FSTL3 overexpression promotes GES1 cell viability and proliferation. (**A**) Overexpression of FSTL3 in GES1 cells monitored using Western blotting; monitoring the housekeeping protein β-actin was used as a loading control. (**B**) Overexpression of FSTL3 in GES1 cells monitored using qRT-PCR. (**C**) Overexpression of FSTL3 in GES1 cells assessed by using the cell viability assay. (**D**) FSTL3 overexpression in GES1 cells was assessed for effects on cell proliferation using EdU incorporation assay. Cell proliferation was determined by EdU staining. (**E**) FSTL3 overexpression and effects on MGC-803 cell colony formation. Error bars indicate +SEM; significance indicated by asterisks, *$P<0.05$, **$P<0.01$, ***$P<0.001$; number of experiments, n=3.

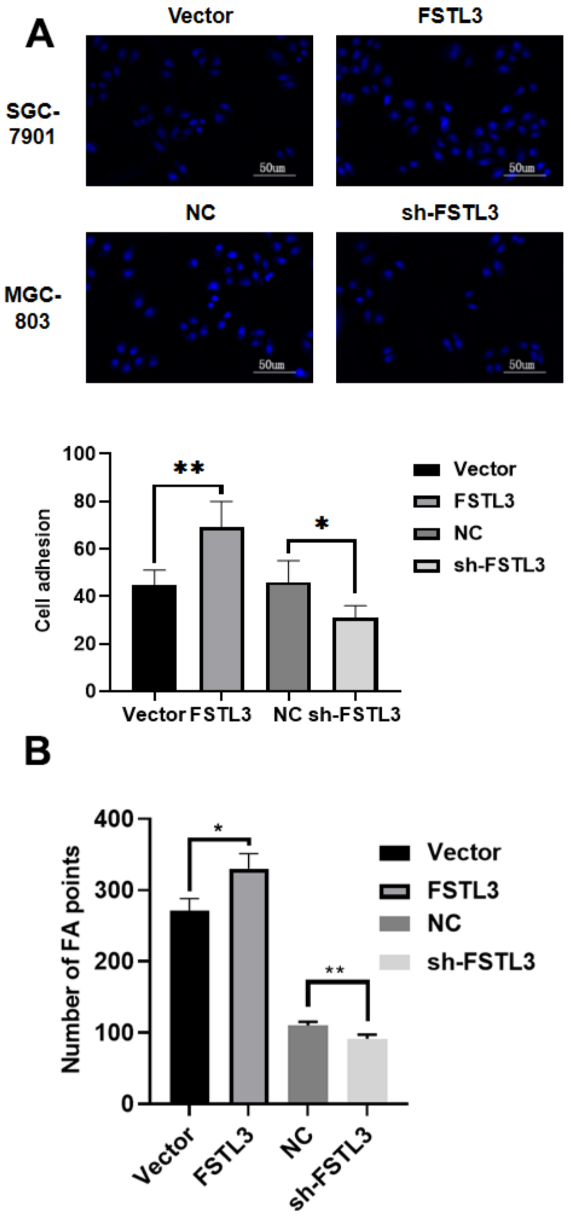

Supplementary Figure 2. FSTL3 overexpression promotes cell adhesion activity and the number of focal adhesion. (**A**) FSTL3 overexpression in MGC-803 cells was assessed for effects on cell adhesion ability. The adhesion ability of MGC-803 cells is expressed as an adhesion ratio. (**B**) FSTL3 overexpression and effects on the number of MGC-803 cell adhesion spots. Error bars indicate +SEM; significance indicated by asterisks, *P<0.05, **P<0.01, ***P<0.001; number of experiments, n=3.
